# Author Correction: Spectroscopy and dynamics of the hydrated electron at the water/air interface

**DOI:** 10.1038/s41467-024-44816-z

**Published:** 2024-01-15

**Authors:** Caleb J. C. Jordan, Marc P. Coons, John M. Herbert, Jan R. R. Verlet

**Affiliations:** 1 Department of Chemistry, Durham University, Durham, DH1 4LJ UK; 2 Department of Chemistry and Biochemistry, The Ohio State University, Columbus, OH USA

**Keywords:** Chemical physics, Electron transfer, Reaction kinetics and dynamics

Correction to: *Nature Communications* 10.1038/s41467-023-44441-2, published online 02 January 2024

In the Acknowledgements section, the funding source ‘EPSRC Doctoral Training Partnership’ should have read ‘Engineering and Physical Sciences Research Council [grant number EP/R513039/1]’. The original article has been corrected.

