# Supplementary material for: Spectroscopy and dynamics of the hydrated electron at the water/air interface
Source: Nat Commun. 2024 Jan 2;15:182. doi: 10.1038/s41467-023-44441-2 (PMC10762076; doi:10.1038/s41467-023-44441-2)
Supplement: Supplementary file 1 — Supplementary Information [file 41467_2023_44441_MOESM1_ESM.pdf]

Supplementary Information for

Spectroscopy and dynamics of the hydrated electron at the water/air interface

Caleb J. C. Jordan,[1] Marc P. Coons,[2] John M. Herbert,[2]* and Jan R. R. Verlet[1]*

Corresponding authors. Email: herbert@chemistry.ohio-state.edu, j.r.r.verlet@durham.ac.uk

Supplementary Note 1:

Fitting procedure

Global fitting was performed on the data shown in Fig. 2(a) as a whole. A global model was proposed to describe the SFG signal, of the form:[5]

$$I_{\mathrm{SFG}}(t, \lambda)^{1/2} = \Sigma_i\, c_i(\lambda)\exp(-t/\tau_i) * G(t)\ ,$$

which is the sum of *i* spectra, $c_i(\lambda)$, that each independently, monoexponentially decay at associated rates τ_i. The model included a convolution with a Gaussian instrument response function $G(t)$, with a width of approximately 250 fs, that was constant across all wavelengths. Using $i = 2$, the above model was fit to all the data in one optimisation, using least-squares fitting (minimizing the absolute error in Solver). The xls sheet is made available together with the data. The fitting was cross-checked using the Levenberg–Marquardt algorithm in (MATLAB), which offered very similar results. The errors are derived from the fitting methods, corresponding to one standard deviation.[5]

Supplementary Note 2:

Consideration of other possible transitions leading to resonance enhancement

Figure 3 assigns the transitions to the hydrated electron and the phenoxyl radical. In principle, the phenoxide anion could also lead to resonance enhancement of the SFG signals. We consider this here. The S_1 and/or S_2 states are initially excited and could be visible in the measurements. The S_1 state has been observed in transient absorption measurements taken following excitation at 266 nm, where it has a lifetime of ~20 ps (from fluorescence measurements).[6] The S_1 excited state absorption is around 515 nm whilst the emission is around 340 nm. Hence, neither are resonant with ω_1, ω_2 or ω_{SFG}. For the S_2 state, the excited state absorption spectrum has, to the best of our knowledge, not been observed.[7,8] This is because the S_2 state lifetime is very short leading to sub-picosecond photo-oxidation to form the phenoxyl radical and electron,[8] as seen in the current measurements. Hence, it seems unlikely that the S_2 state will contribute to the SFG signal. Finally, from a dynamical perspective, we would anticipate that if the spectrum peaking around 720 in Figure 3 was arising from resonance enhancement from the S_2 (or S_1) state of phenolate, then its dynamics would be correlated with those of the phenoxyl radical, but they are not (Figure 2). We conclude that the most likely assignment is that the short-lived species arises from the interfacial hydrated electron and the long-lived component from the phenoxyl radical.

Consideration of electronic SFG spectrum's correspondence to the absorption spectrum

The electronic SFG spectra are expected to closely resemble the bulk absorption spectra on account of both measurements being proportional to the resonant transition dipole moment (TDM). For the case of a singly-resonant fundamental field, ω_1, that induces the transition $n \leftarrow g$, the SFG signal contribution can be written as:[9,10]

$$\chi^{(2)} \propto \frac{\mu_{gn'}\mu_{n'n}\mu_{ng}}{\Omega_{ng} - \omega_1 - i\Gamma_{ng}} \ .$$

In the above expression, μ_{ng} is the TDM for the transition $n \leftarrow g$, and state n' is the virtual state accessed by $\omega_1 + \omega_2$ or ω_{SFG} from state g. Ω_{ng} is the resonant frequency of the $n \leftarrow g$ transition, and Γ_{ng} is its width.

In the case of $e_{(aq)}^{-}$, the primary resonance term is associated with ω_1, where g is the 1s state and n the 1p state(s). The terms $\mu_{gn'}$ and $\mu_{n'n}$ are broadly nonresonant, in which case they are expected to be approximately constant in the wavelength regions probed in the

experiment. Hence, we might anticipate that the electronic SFG spectrum will be approximately proportional to the absorption spectrum.

In the case of the phenoxyl radical, where ω_{SFG} is resonant (as well as the sum $\omega_1 + \omega_2$), n' corresponds to an excited state of the molecule, the C^2B_1 excited state, and therefore n is a virtual state. In this scenario, the $\chi^{(2)}$ becomes proportional to the two-photon TDM as well as that of the one-photon TDM. However, in general, a two-photon TDM is very small (especially for a small molecule with small hyperpolarizability) and the associated two-photon spectrum tends to be broader than the single-photon absorption. Hence, in the wavelength range considered here, the two-photon term is likely to be small and approximately constant so that the signal will predominantly follow the $C^2B_1 \leftarrow X^2B_1$ one-photon absorption spectrum.

To further support that the absorption spectra generally follow the linear absorption spectra, there have been a few studies comparing surface electronic SFG spectra with absorption spectroscopy (as well as many comparing vibrational SFG spectra with IR spectroscopy), which illustrate the often close agreement between the two. In general, these have relied on Malachite green as a test case. Sen *et al.* showed a close correspondence between the electronic SFG spectrum and the absorption spectrum for the $S_2 \leftarrow S_0$ transition using broadband electronic SFG.[11] Qian *et al.* similarly showed the $S_1 \leftarrow S_0$ transition to be well captured by the electronic SFG spectrum.[12] In both cases, the difference in spectral maximum between SFG and UV-vis was less than 10 nm.

Consideration of the probing depth of the SFG signal

Firstly, because the time-resolved SFG experiments are clearly different to bulk transient absorption data,[7] it seems unlikely that bulk dynamics are observed. Nevertheless, we consider here other possibilities. In principle, symmetry can be broken over an extended range because of bi-layer formation. Phenoxide has some surface affinity (although much weaker than phenol) and there is a significant (150 mM) concentration of sodium ions in the solution (due to the addition of NaOH to bring the solution to pH 13). The effect of concentration and pH was be probed by comparing previous measurements taken with ω_1 at $\lambda = 720$ nm[13] to those presented here at the same ω_1. Reducing the pH from 13 to 12 or reducing the concentration from 150 mM to 100 mM showed no noticeable effect on the dynamics.

We also briefly contrast the current work with our previous second-harmonic generation (SHG) work following charge-transfer-to-solvent (CTTS) excitation of iodide at the water/air interface.[14,15] The hydrated electron was observed in these experiments with its kinetics appearing similar to those in the bulk and remaining visible for 100s ps, in contrast to current observations. However, the concentration was much higher (2 M NaI) and, according to molecular dynamics simulations, at such concentrations, the range over which symmetry is broken extends at least 1 nm.[16] Indeed, this conclusion is consistent with experiments in which surfactants were added to the solution.[14] The fact that hydrated electrons are observed for 100s ps in the SHG experiments for concentrated NaI solutions following CTTS excitation therefore suggests that the electrons remains within ~1 nm of the interface. Hence, the larger probe depth accessible in these experiments can account for the longer observation time. Alternatively, the very large concentration of counter ions (Na^+) might be driving the electrons to the interface. We intend to probe the effect of counter-ions in the current experiments in due course.

Consideration of symmetry

$\chi^{(2)}$, as probe in electronic SFG, is a macroscopic observable of the time- and orientationally averaged molecular hyperpolarisabilities of the surface, β. In the case of the hydrated electron, which in the bulk has a pseudo-spherically-symmetric structure, we expect the structure near the interface to be perturbed by the interfacial symmetry. More specifically, we expect the orbitals that protrude into/out of the interfacial layer, which lie along the vertical laboratory axis, to be the most affected, given they experience the largest changes in field gradients and solvent density along their length. Hence, for SFG fields in the PPP configuration that are incident at approximately 73° to the surface normal, the vertical surface component will be the largest contribution to the measured signal ($\chi_{zzz}^{(2)}$). The components of $\chi^{(2)}$ parallel to the plane of the surface are expected to have properties closer to the bulk hydrated electron, but these are probed more weakly.

Supplementary Note 3:

Consideration of vacuum as a model for the air phase.

The simulations consider the air-phase as a vacuum. This is a reasonable assumption in the current simulations and experiments. At 1 atm pressure and 300 K, the collision rate for a gas-phase molecule (be it N_2 or O_2) per unit area is

$$z = \tfrac{1}{4} N V^{-1} \langle v \rangle = \tfrac{1}{4} \times 2.43 \times 10^{25}\ \mathrm{m^{-3}} \times 440\ \mathrm{m\ s^{-1}} = 2.70 \times 10^{27}\ \mathrm{s^{-1}\ m^{-2}}.$$

The effective area of the hydrated electron using $r_g = 2.5 \times 10^{-10}$ m is 1.96×10^{-19} m^2 so that the collision rate of an atmospheric molecule with the hydrated electron at the water/air interface is approximately 5×10^{8} s^{-1} corresponding to a collision every 2 ns on average. This timescale exceeds both the simulation and the experiment sufficiently so that the vapor phase can be viewed as vacuum.

Supplementary Note 4:

Surface excess from surface tension measurements

Both phenol and phenolate are surface active. The following figures were obtained by performing surface tensiometry measurements by pendant drop-shape analysis, and fitting to data using the Szyszkowski equation. The data are in very good agreement with previous literature.[17]

Supplementary Figure 1: Surface tensiometry measurements for phenol.

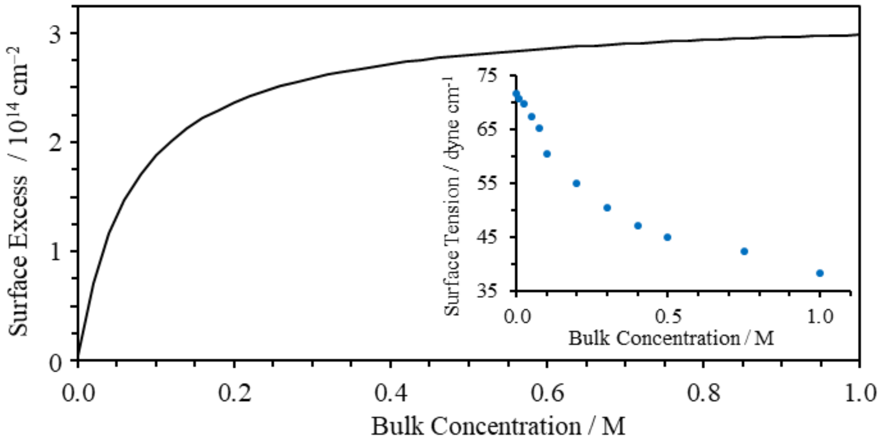

Supplementary Figure 2: Surface tensiometry measurements for phenolate.

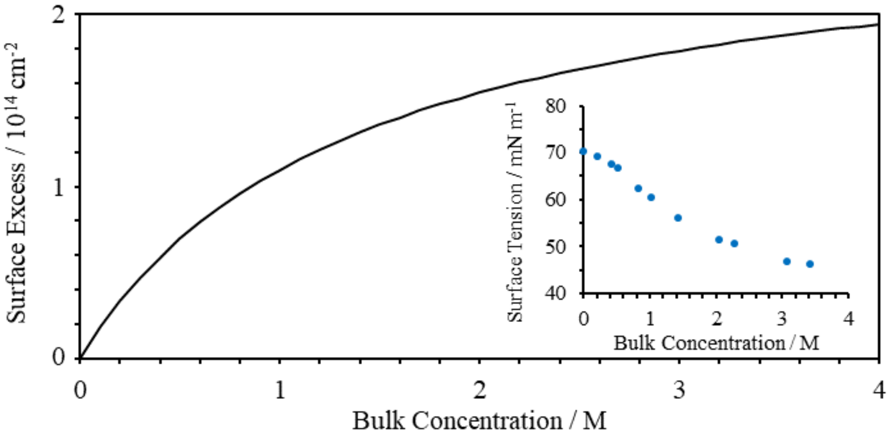

Supplementary References

(1) Jordan, C. J. C.; Verlet, J. R. R. Time-Resolved Electronic Sum-Frequency Generation Spectroscopy with Fluorescence Suppression Using Optical Kerr Gating. *J. Chem. Phys.* **2021**, *155* (16), 164202. https://doi.org/10.1063/5.0065460.

(2) Coons, M. P.; You, Z.-Q.; Herbert, J. M. The Hydrated Electron at the Surface of Neat Liquid Water Appears To Be Indistinguishable from the Bulk Species. *J. Am. Chem. Soc.* **2016**, *138* (34), 10879–10886. https://doi.org/10.1021/jacs.6b06715.

(3) Turi, L.; Borgis, D. Analytical Investigations of an Electron–Water Molecule Pseudopotential. II. Development of a New Pair Potential and Molecular Dynamics Simulations. *The Journal of Chemical Physics* **2002**, *117* (13), 6186–6195. https://doi.org/10.1063/1.1503308.

(4) Jacobson, L. D.; Herbert, J. M. A One-Electron Model for the Aqueous Electron That Includes Many-Body Electron-Water Polarization: Bulk Equilibrium Structure, Vertical Electron Binding Energy, and Optical Absorption Spectrum. *J. Chem. Phys.* **2010**, *133* (15), 154506. https://doi.org/10.1063/1.3490479.

(5) *Measurements and their Uncertainties - Ifan Hughes, Thomas Hase - Oxford University Press*. https://global.oup.com/ukhe/product/measurements-and-their-uncertainties-9780199566334 (accessed 2023-10-09).

(6) Chen, X.; Larsen, D. S.; Bradforth, S. E.; van Stokkum, I. H. M. Broadband Spectral Probing Revealing Ultrafast Photochemical Branching after Ultraviolet Excitation of the Aqueous Phenolate Anion. *J. Phys. Chem. A* **2011**, *115* (16), 3807–3819. https://doi.org/10.1021/jp107935f.

(7) Tyson, A. L.; Verlet, J. R. R. On the Mechanism of Phenolate Photo-Oxidation in Aqueous Solution. *J. Phys. Chem. B* **2019**, *123* (10), 2373–2379. https://doi.org/10.1021/acs.jpcb.8b11766.

(8) Robertson, K.; Fortune, W. G.; Davies, J. A.; Boichenko, A. N.; Scholz, M. S.; Tau, O.; Bochenkova, A. V.; Fielding, H. H. Wavelength Dependent Mechanism of Phenolate Photooxidation in Aqueous Solution. *Chem. Sci.* **2023**, *14* (12), 3257–3264. https://doi.org/10.1039/D3SC00016H.

(9) Richmond, G. L. Molecular Bonding and Interactions at Aqueous Surfaces as Probed by Vibrational Sum Frequency Spectroscopy. *Chem. Rev.* **2002**, *102* (8), 2693–2724. https://doi.org/10.1021/cr0006876.

(10) Yamaguchi, S.; Tahara, T. Precise Electronic χ(2) Spectra of Molecules Adsorbed at an Interface Measured by Multiplex Sum Frequency Generation. *J. Phys. Chem. B* **2004**, *108* (50), 19079–19082. https://doi.org/10.1021/jp045306x.

(11) Sen, P.; Yamaguchi, S.; Tahara, T. Ultrafast Dynamics of Malachite Green at the Air/Water Interface Studied by Femtosecond Time-Resolved Electronic Sum Frequency Generation (TR-ESFG): An Indicator for Local Viscosity. *Faraday Discuss.* **2010**, *145* (0), 411–428. https://doi.org/10.1039/B908097J.

(12) Qian, Y.; Deng, G.; Rao, Y. In Situ Spectroscopic Probing of Polarity and Molecular Configuration at Aerosol Particle Surfaces. *J. Phys. Chem. Lett.* **2020**, *11* (16), 6763–6771. https://doi.org/10.1021/acs.jpclett.0c02013.

(13) Jordan, C. J. C.; Lowe, E. A.; Verlet, J. R. R. Photooxidation of the Phenolate Anion Is Accelerated at the Water/Air Interface. *J. Am. Chem. Soc.* **2022**, *144* (31), 14012–14015. https://doi.org/10.1021/jacs.2c04935.

(14) Sagar, D. M.; Bain, C. D.; Verlet, J. R. R. Hydrated Electrons at the Water/Air Interface. *Journal of the American Chemical Society* **2010**, *132* (20), 6917-+. https://doi.org/10.1021/ja101176r.

(15) Nowakowski, P. J.; Woods, D. A.; Verlet, J. R. R. Charge Transfer to Solvent Dynamics at the Ambient Water/Air Interface. *J. Phys. Chem. Lett.* **2016**, *7* (20), 4079–4085. https://doi.org/10.1021/acs.jpclett.6b01985.

(16) Jungwirth, P.; Tobias, D. J. Ions at the Air/Water Interface. *J. Phys. Chem. B* **2002**, *106* (25), 6361–6373. https://doi.org/10.1021/jp020242g.

(17) Rao, Y.; Subir, M.; McArthur, E. A.; Turro, N. J.; Eisenthal, K. B. Organic Ions at the Air/Water Interface. *Chemical Physics Letters* **2009**, *477* (4), 241–244. https://doi.org/10.1016/j.cplett.2009.07.011.
